# Elective lower limb orthopedic arthroplasty surgery in patients with pulmonary hypertension

**DOI:** 10.1002/pul2.12019

**Published:** 2022-03-25

**Authors:** Mikaela Wardle, Amanda Nair, Sarah Saunders, Iain Armstrong, Athanasios Charalampopoulos, Charlie Elliot, Abdul Hameed, Neil Hamilton, John Harrington, Carol Keen, Robert Lewis, Ian Sabroe, A. A. Roger Thompson, Robert M. Kerry, Robin Condliffe, David G. Kiely

**Affiliations:** ^1^ Sheffield Pulmonary Vascular Disease Unit Sheffield Teaching Hospitals NHS Trust Sheffield UK; ^2^ Department of Anaesthetics Sheffield Teaching Hospitals NHS Trust Sheffield UK; ^3^ Department of Infection, Immunity and Cardiovascular Disease University of Sheffield Sheffield UK; ^4^ Department of Orthopaedic Surgery Sheffield Teaching Hospitals NHS Trust Sheffield UK

**Keywords:** anaesthesia, chronic thromboembolic pulmonary hypertension, perioperative management, pulmonary arterial hypertension

## Abstract

Patients with pulmonary arterial hypertension and chronic thromboembolic pulmonary hypertension (PH) are at increased risk when undergoing anesthesia and major surgery. Data on outcomes for elective orthopedic surgery in patients with PH are limited. A patient pathway was established to provide access to elective lower limb arthroplasty. This included assessment of orthopedic needs, fitness for anesthesia, preoperative optimization, and intra‐ and postoperative management. Patient data were retrospectively retrieved using patient's hospital records. Between 2012 and 2020, 29 operations (21 total hip replacements [THRs], 7 total knee replacements [TKRs], 1 total hip revision) were performed in 25 patients (mean age: 67 years). Perioperatively, 72% were treated with low‐dose intravenous prostanoid. All had arterial lines, and central access and perioperative lithium dilution cardiac output monitoring was used in 86% of cases. Four patients underwent GA, 21 spinal anesthesia, and 4 CSE anesthesia. Supplemental nerve blocks were performed in all patients undergoing general, and 12 of 21 undergoing spinal anesthesia. All were managed in high dependency postoperatively. Hospital length of stay and complication rates were higher than reported in non‐PH patients. Perioperative complications included hypotension requiring vasopressors (*n* = 10), blood transfusion (*n* = 7), nonorthopedic infection (*n* = 4), and decompensated right heart failure (*n* = 1). There was no associated mortality. All implants were functioning well at 6 weeks and subsequent follow‐up. EmPHasis‐10 quality of score decreased by 5.5 (±2.1) (*p* = 0.04). A dedicated multiprofessional pathway can be used to safely select and manage patients with PH through elective lower limb arthroplasty.

## INTRODUCTION

Pulmonary hypertension (PH) is comprised of a heterogeneous group of conditions ranging from rare diseases such as pulmonary arterial hypertension (PAH) and chronic thromboembolic pulmonary hypertension (CTEPH) to more common, and usually milder, elevations in pulmonary artery pressure seen in cardiac and respiratory disease.^1^ Advances in available therapies over the last two decades have resulted in improved survival of patients with PAH and CTEPH^2^, ^3^, ^4^ and as such there has been an increasing focus on quality of life.^5^ Osteoarthritis (OA) is an age‐related degenerative joint disease, affecting 11% of people in England.^6^ It causes progressive damage to articular cartilage and surrounding structures and most commonly affects the hip and knee.^6^ It is the fastest‐growing cause of disability worldwide and is often associated with constant severe pain, reduced quality of life, and economic burden.^6^, ^7^, ^8^ The definitive treatment for severe hip and knee OA is arthroplasty surgery.^9^ In patients without the cardiorespiratory disease, elective hip and knee arthroplasty is a cost‐effective, low‐risk procedure with high success rates.^9^, ^10^, ^11^

Patients with PH, particularly those with PAH and CTEPH, are at increased risk when undergoing anesthesia and major surgery.^12^ Major, prolonged and emergency surgery has been associated with increased morbidity, and better outcomes have been associated with regional anesthesia compared to general anesthesia.^13^, ^14^, ^15^, ^16^, ^17^, ^18^ Perioperative mortality rates have reported to vary between 1% and 18%.^13^, ^14^, ^15^, ^16^, ^17^, ^19^, ^20^ To our knowledge, only one study has exclusively evaluated the perioperative mortality rate in patients with PH undergoing total hip or knee replacement surgery compared to those without PH. In this study, Memtsoudis et al. demonstrated a 4 to 4.5‐fold increase in the adjusted mortality risk compared to patients without PH in a US database of 670,515 patients undergoing total hip or knee arthroplasty.^20^ Price et al. reported a 7% mortality in 28 patients with mild to moderate PH undergoing nonobstetric and noncardiac surgery, with no disease deterioration in surviving patients when assessed at 3–6 months after surgery. They concluded that nonemergency procedures may not be contraindicated in patients with PH if they are carefully selected and managed in a specialist PH center.^13^, ^15^

In this study, we report outcomes from a prospective pathway established by a multiprofessional team to enable access for patients with PAH and CTEPH to elective lower limb orthopedic arthroplasty.

## METHODS

### Setting

We performed a single‐center retrospective study of patients with PAH and CTEPH undergoing elective lower limb orthopedic surgery via a dedicated pathway, including detailed preoperative assessment. All patients were managed at the Sheffield Pulmonary Vascular Disease Unit (PVDU), which is a referral center for the assessment and management of patients with PH with a referral population >15 million. Patients underwent systematic evaluation as described in the ASPIRE Registry including right heart catheterization (RHC), multimodality imaging, exercise, and lung function testing.^2^

### Data collection

All PH patients undergoing orthopedic surgery between December 2010 and January 2020 were identified. Patient characteristics, pulmonary hemodynamics, results of radiological investigations, therapies, and details of the referral process were obtained from hospital notes and databases. Anesthetic and operative data were obtained from the preoperative assessment and intraoperative anesthetic and operation notes. Perioperative outcomes were retrieved from inpatient records. Postoperative orthopedic and quality of life (QoL) outcomes were assessed from orthopedic clinic notes 6 weeks after surgery and by comparing the last preoperative and first postoperative emPHAsis‐10 score (a PH‐specific QoL tool) documented in patient's notes.^21^ The census date for mortality was August 18, 2021.

### Statistical analysis

The Shapiro–Wilk test was used to determine if the data were normally distributed. Normal distribution was assumed for all data which returned a *p *> 0.05. Data were also inspected via graphical methods (Q‐Q Plots and Histograms). Paired sample *t* tests were performed on diagnostic and preoperative data where two observations from the patients had been collected. Data were presented as mean ± standard deviation or median (range). A *p* < 0.05 was considered statistically significant.

## RESULTS

### Preoperative assessment

**Figure 1:**
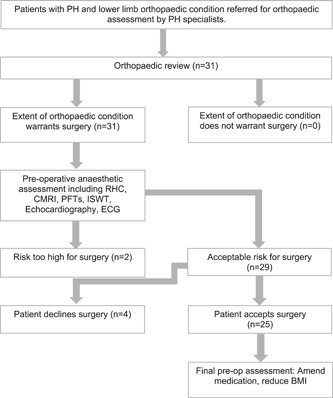
Preoperative patient pathway. CMRI, cardiac magnetic resonance imaging; ECG, electrocardiograph; ISWT, incremental shuttle walking test; PFTs, pulmonary function tests; RHC, right heart catheterization

During the study period, 31 patients with lower limb orthopedic problems were referred for orthopedic assessment (Figure 1). All patients were deemed suitable surgical candidates from the orthopedic perspective pending further cardiopulmonary and anesthetic assessment. To be considered a suitable candidate for orthopedic surgery one or more of the following were required: constant severe pain at rest and at night, pain not responding to conservative treatment and a significant restriction in mobility negatively impacting quality of life.

The 31 patients were then electively admitted to the Sheffield PVDU for a detailed operative and anesthetic assessment with investigations including RHC, cardiac MRI, echocardiography, ECG, pulmonary function tests, and exercise testing using the incremental shuttle walk test (ISWT) (Figure 1). Twenty‐nine patients were considered to have an acceptable medical risk, while two patients were deemed to be too high risk. In one case, this was due to the severity of their PH and estimated life expectancy, while in the second case the risks of surgery were felt to be prohibitive due to the presence of significant comorbidities. Three patients in the acceptable medical risk group decided against surgery following counseling regarding the risks and benefits of surgery. One patient decided not to proceed with surgery after their symptoms improved with further steroid injections between orthopedic and anesthetic assessment. Therefore, 25 patients decided to proceed with surgery and underwent final preoperative assessments to finalize a perioperative management plan with a consultant anesthetist and PH specialist following multidisciplinary assessment. Four patients had a subsequent second‐sided operation meaning that 29 cases in total were performed.

### Patient demographics, hemodynamics, and functional status

Twenty‐nine elective lower limb operations were carried out on 25 patients; baseline characteristics are shown in Table 1. Twenty‐four (96%) patients were female. All patients were in World Health Organisation Functional Class II or III at the time of their operation. All were categorized in American Association Anaesthesiologists Group 3 or 4.^22^ Forty‐one percent of patients had been established on oral monotherapy, 48% on oral combination therapy, and 3% on combination therapy involving inhaled iloprost. After a mean interval of 3.3 ± 2.93 years between diagnostic and preoperative RHC, significant improvements in mean pulmonary artery pressure (mPAP), pulmonary vascular resistance, and mixed venous saturations were observed (Table 1). Patients had moderate PH at the time of surgery with a mean mPAP of 37.2 ± 10.2 mmHg (10.2) and cardiac output 4.9 ± 1.5 L/min. The majority of patients had preserved or mildly impaired right ventricular (RV) function, Table 1.

**Table 1:** Baseline characteristics

Patient number	25
Gender (% [number] female)	96 (24)
Age (years)	66.9 ± 13
BMI (kg/m^2^)^a^	26 ± 5
PH Type (% [number])	
Idiopathic PAH	24 (6)
CTD‐PAH	32 (8)
CHD‐PAH	8 (2)
Portopulmonary	8 (2)
CTEPH (inoperable)	8 (2)
CTEPH (residual)	16 (4)
Combined pre‐ and postcapillary PH	4 (1)
Diagnostic RHC	
mRAP (mmHg)	9.9 ± 5.1
mPAP (mmHg)	42.9 ± 11.6
PAWP (mmHg)	11.6 ± 3.5
CO (L/min)	5.0 ± 1.6
CI (L/min/m^2^)	2.9 ± 0.9
PVR (WU)	7.8 ± 4.7
sVO_2_(%)	63.7 ± 15.6
Preoperative RHC^b^	
mRAP (mmHg)	7.3 ± 5.0
mPAP (mmHg)	37.2 ± 10.2*
PAWP (mmHg)	10.6 ± 4.7
CO (L/min)	4.9 ± 1.5
CI (L/min/m^2^)	2.9 ± 1.0
PVR (WU)	6.1 ± 3.5*
sVO_2_(%)	70.4 ± 7.1*
Preoperative RV function (% [number])^d^	
Poor	3 (1)
Moderately impaired	3 (1)
Mildly impaired	55 (16)
Preserved	38 (11)
Preoperative LV function (% [number])^c^, ^e^	
Poor	0 (0)
Moderately impaired	0 (0)
Mildly impaired	4 (1)
Preserved	96 (26)
ASA grade (% [number])^d^	
3	62 (18)
4	38 (11)
WHO FC (%)^d^	
II	28 (8)
III	72 (21)

*Note*: Data presented as number, % or mean (±standard deviation). Where percentages are displayed, numbers may not sum to 100 due to rounding.

Abbreviations: ASA, American Society of Anesthesiologists Classification; BMI, body mass index; CHD, congenital heart disease; CI, cardiac index; CO, cardiac output; CTD, connective tissue disease; CTEPH, chronic thromboembolic pulmonary hypertension; LV, left ventricular; mPAP, mean pulmonary arterial pressure; mRAP, mean right atrial pressure; PAH, pulmonary arterial hypertension; PAWP, pulmonary arterial wedge pressure; PH, pulmonary hypertension; PVR, pulmonary vascular resistance; RHC, right heart catheterization; sVO2, mixed venous saturations; WHO FC, World Health Organisation Functional Class; WU, Wood Units; RV, right ventricular.

^a^ At the time of first surgery.

^b^ Mean 10.2 months between RHC and surgery. Based on 22 cases, a cardiac MRI was carried out before operation in the case of the other seven instances in place of repeat RHC.

^c^ Cardiac MRI in 24 patients and echocardiography in five patients.

^d^ Data presented based on 29 cases.

^e^ Based on 27 cases as LV function not reported on two MRI scans.

* *p* < 0.05 at paired *t* test compared with baseline.

### Perioperative PH therapies

Twenty‐one (72%) cases were admitted 48–72 h before surgery to commence a low‐dose intravenous iloprost (Ilomedin) infusion (dose range: 1–3 μg/h), which was then continued intraoperatively and in to the immediate postoperative period for a maximum of 5 days. Baseline oral PH therapy was continued in all cases. The patients who did not receive preoperative intravenous iloprost all had had stable disease with preserved or mildly impaired RV function.

### Operative details

**Figure 2:**
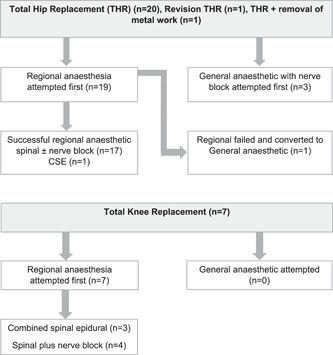
Anesthetic technique

Twenty total hip replacements (THRs), seven total knee replacements (TKRs), one THR with the removal of metalwork, and one total hip revision were carried out in 25 patients (Figure 2). Four patients had a second THR on the contralateral joint following a good outcome from the first operation. Uncemented implants were used for all THRs, whereas cemented implants were used for all TKRs. An intraoperative tourniquet was used for all TKR procedures. All operations were carried out by the same orthopedic surgeon (R. K.).

### Anesthetic technique

Regional anesthesia was the preferred option for all procedures (Tables 2 and 3). For THR, spinal anesthesia with intrathecal diamorphine was the preferred regional anesthetic technique, being used in 18/22 hip procedures. Spinal anesthetics were supplemented with either local anesthetic infiltration by the surgeon, fascia iliaca block, or femoral nerve blocks to improve postoperative analgesia. General anesthesia (GA) was used in four THR operations, in two patients. One of these patients had a failed spinal for their first procedure, and so had a GA for their second operation, because of this previous failure. The second patient had a GA for both operations, due to their personal choice. All four GA cases were supplemented with either femoral nerve block or fascia iliaca block for postoperative analgesia. Regional anesthesia was performed for all TKR operations. For four patients, this was provided in the form of a spinal anesthetic with additional nerve block (either saphenous nerve/adductor canal block alone or in combination with a popliteal nerve block). In the remaining three TKR operations a combined spinal‐epidural (CSE) was used to provide further postoperative analgesia. The initial two TKR operations were performed with spinal and nerve blocks, and both experienced severe postoperative pain and subsequent cardiorespiratory issues. Following multidisciplinary team discussion with the acute pain, critical care, and anesthetic teams, a CSE technique was chosen for TKR patients who were considered likely to experience more severe postoperative pain. For the patients who underwent general anesthesia, induction was performed with propofol (range: 60–80 mg) and fentanyl (100–150 μg); the patient who was given a GA because of the failed spinal was not given fentanyl.

**Table 2:** Anesthetic data for patients who underwent general anesthesia

Operation	Reason for GA	Additional regional used	Airway device	Induction opiate	Other induction	Muscle relaxant	Anesthetic maintenance	Ventilation Mode	Additional analgesia	Iloprost intraoperative	Metaraminol^a^ intraoperative	Noradrenaline intraoperative	Intraoperative monitoring
THR	Previous failed spinal	Femoral block	LMA	Fentanyl 75 μg	Propofol 80 mg	Not used	Sevoflurane, oxygen, air	Spont + PS	Morphine 7 mg, paracetamol 1 g	Not used	Intermittent boluses	Not required	A‐line, CVC, LiDCO
THR	Spinal performed pain on incision	Spinal + Fascia iliaca block	LMA	Not used	Propofol 100 mg	Not used	Sevoflurane, oxygen, air	Spont + PS	Unclear	Not used	8 ml/h	Not required	A‐line, CVC, LiDCO
THR	Patient preference	Fascia iliaca block	ETT	Fentanyl 100 μg	Propofol 60 mg	Cisatracurium 12 mg	Sevoflurane, oxygen, air	VC, TV 500 ml, RR 10, No PEEP	Morphine 8 mg, paracetamol 1 g	1 mcg/h	Intermittent boluses	Not required	A‐line, CVC, LiDCO
THR	Patient preference	Fascia iliaca block	ETT	Fentanyl 150 μg	Propofol 80 mg	Rocoruniom 60 mg	Sevoflurane, oxygen, air	PCV	unclear	Yes (dose unclear)	1 ml/h	Not required	A‐line, CVC, LiDCO

Abbreviations: A‐line, arterial line; CVC, central venous catheter; ETT, endotracheal tube; GA, general anesthetic; intra‐op, intra‐operatively; LiDCO, lithium dilution cardiac output; LMA, laryngeal mask airway; PEEP, positive end‐expiratory pressure; PVC, pressure‐controlled ventilation; Spont. + PS, spontaneous ventilation plus pressure support ventilation; THR, total hip replacement; TV, tidal volume; VC, volume‐controlled ventilation

^a^ Metaraminol was given as an infusion at a concentration of 0.5mg/ml.

**Table 3:** Anesthetic data for patients who underwent regional anesthesia

Procedure	Regional technique	Intraoperative monitoring	Iloprost intraoperative	Metaramino^b^ intraoperative	Noradrenaline intraoperative
THR	Spinal^a^	A‐line, CVC, Lidco	Not used	Yes (dose unclear)	Not required
THR	Spinal	A‐line, CVC, Lidco	3 mcg/h	2 ml/h	Not required
THR	Spinal	A‐line, CVC	Not used	Not used	Not required
THR	Spinal	A‐line, Lidco, CVC	Not used	Yes (dose unclear)	Not required
THR	Spinal	A‐line, CVC, Lidco	2 mcg/h	6 ml/h	Not required
THR	Spinal	A‐line, CVC, Lidco	2 mcg/h	8 ml/h	Not required
THR	Spinal	A‐line, CVC, Lidco	Yes (dose unclear)	7 ml/h	Not required
THR	Spinal	A‐line, CVC, Lidco	2 mcg/h	5 ml/h	Not required
THR	Spinal + fascia iliaca block	A‐line, CVC, Lidco	3 mcg/h	5 ml/h	Not required
THR	Spinal + fascia iliaca block	A‐line, CVC, Lidco	Not used	Not used	Not required
THR	Spinal + fascia iliaca block	A‐line, CVC, Lidco	2 mcg/h	1 ml/h	Not required
THR	Spinal + fascia iliaca block	A‐line, CVC, Lidco	Yes (dose unclear)	4 ml/h	Not required
THR	Spinal + fascia iliaca block	A‐line, CVC, Lidco	2 mcg/h	Not used	Not required
THR	Spinal + fascia iliaca block	A‐line, CVC, Lidco	3 mcg/h	3 ml/h	Not required
THR	Spinal + fascia iliaca block	A‐line, CVC	2 mcg/h	Not used	Not required
THR	Spinal + femoral block	A‐line, CVC, Lidco	3 mcg/h	4 ml/h	Not required
THR and removal of metalwork	Spinal	A‐line, CVC, Lidco	3 mcg/h	0.5 ml/h	Not required
Revision THR	CSE + torniquet	A‐line, CVC, Lidco	3 mcg/h	5 ml/h	Not required
TKR	Spinal + femoral block	A‐line, CVC	Yes (dose unclear)	Not used	0.05 μg/kg/min
TKR	Spinal + saphenous and popliteal block	A‐line, CVC, Lidco	2 mcg/h	Not used	Not required
TKR	Spinal + adductor canal block	A‐line, CVC, Lidco	Not used	4 ml/h	Not required
TKR	CSE + torniquet	A‐line, CVC	2 mcg/h	6 ml/h	Not required
TKR	CSE + torniquet	A‐line, CVC, Lidco	3 mcg/h	4 ml/h	Not required
TKR	CSE + torniquet	A‐line, CVC, Lidco	Not used	8 ml/h	Not required
TKR	Spinal + adductor canal + L popliteal block	A‐line, CVC, Lidco	2 mcg/h	5 ml/h	Not required

Abbreviations: A‐line, arterial line; CSE, combined spinal‐epidural anesthetic; CVC, central venous catheter; GA, general anesthetic; intra‐op, intra‐operatively; LiDCO, lithium dilution cardiac output; THR, total hip replacement; TKR, total knee replacement

^a^ All spinals were with bupivacaine 0.5% plus diamorphine.

^b^ Metaraminol was given as an infusion at a concentration of 0.5mg/ml.

A general principle was to avoid positive pressure ventilation where possible. Two GAs were managed with the patient breathing spontaneously via a supraglottic airway device (SAD), with minimal pressure support ventilation to maintain a normal carbon dioxide level. The other two patients were intubated and were given a nondepolarizing muscle relaxant.

Anesthesia was maintained with oxygen, air, and sevoflurane for all GA cases. Volume‐controlled ventilation was used in one patient who was intubated, pressure control ventilation was used in the other patient who was intubated and had a background of bronchiectasis in addition to PH to avoid high airway pressures.

### Intraoperative monitoring and support

All patients were monitored with arterial line and central venous access monitoring in addition to standard requirements. All patients were catheterized to measure urine output. In 25/29 cases, lithium dilution cardiac output monitoring (LiDCO) was used to enable a real‐time and continuous assessment of cardiac output and goal‐directed fluid therapy. A metaraminol infusion was used in 76% of all cases where intravenous iloprost pre‐operatively had been started (iloprost was started preoperatively in 72% cases). One patient required intraoperative noradrenaline. Anesthetic management was provided by one of two anesthetists.

### Postoperative care

All patients were extubated in theatre and received Level 2 postoperative care on a high dependency unit. In 26 out of 29 cases, patients were stepped down to a specialized PH ward. All patients received daily review from the PH, orthopedic, and physiotherapy teams. There was no perioperative mortality.

### Physiotherapy

Patients were able to start physiotherapy after a mean time of 3 days following surgery. The mean time to complete physiotherapy was 6 days. Fifty‐five percent of patients were deemed as “slow to mobilise” by the orthopedic physiotherapists and required 6 or more days of in‐patient physiotherapy. Twenty‐three percent of patients required further intensive physiotherapy in the community after discharge.

### Complications

In 21 out of 29 cases (72%), patients experienced one or more complications in the immediate postoperative period. The most common complication was hypotension requiring vasopressor support immediately after surgery (34%), which was weaned over a mean time of 3 days. Other complications included blood loss requiring transfusion (24%), significant pain requiring additional opiates or hindering physiotherapy (10%), and lower respiratory tract infection requiring antibiotics (14%). No patients required readmission to critical care after discharge to the ward. All patients survived and were discharged home after a mean hospital stay of 13 nights. One patient, receiving hydroxychloroquine for a connective tissue disease, was readmitted shortly after discharge with fever due to CMV viremia. One patient, with systemic sclerosis, presented 2 months after surgery with occlusion of the radial artery which had undergone arterial cannulation and required a finger amputation.

### Orthopedic outcomes

All patients attended their 6‐week postoperative orthopedic review. One patient had developed a noninfective wound leak. No other complications were noted. All joints were reported as “functioning well” or “making good progress” with all patients reporting an improvement in pain. All five patients who used walking aids before their operation (sticks, wheelchair, or electric scooter outside) reported a reduction in their use. Nineteen (76%) patients were alive at the census date of August 18, 2021. Median (range) survival from date of surgery of the 6 patients who subsequently died was 24 months.^9^, ^10^, ^11^, ^12^, ^13^, ^14^, ^15^, ^16^, ^17^, ^18^, ^19^, ^20^, ^21^, ^22^, ^23^, ^24^, ^25^, ^26^, ^27^, ^28^, ^29^, ^30^, ^31^, ^32^, ^33^, ^34^

### Quality of life

We investigated the impact of surgery on QoL by comparing patients' last documented preoperative and first documented postoperative emPHAsis‐10 score. Paired data were available in 17 cases with 14/17 patients reporting either no change or improved quality of life with a mean (SD) score decrease of 5.5 (±2.1) (*p* = 0.04).

## DISCUSSION

Over a 5‐year period, a dedicated pathway for elective orthopedic surgery enabled 25 patients with PH to undergo 29 elective lower limb arthroplasty with good short‐term outcomes and improved quality of life. To our knowledge, this is the first study to primarily focus on a systematic multiprofessional approach to the provision of lower limb arthroplasty surgery in patients with PAH or CTEPH, with a focus on preoperative evaluation and patient selection, optimization of PH treatment, perioperative monitoring, and outcome.

### Short‐term outcomes and comparison with other studies

Our pathway has demonstrated lower mortality rates (0%) than previous studies evaluating the outcomes of noncardiac, nonobstetric surgery in patients with PAH. This may reflect, in part, patient selection, the nature of the surgery, and careful perioperative management by a dedicated multiprofessional team. Memtsoudis et al. analyzed a large US in‐patient database and observed perioperative mortality in patients with idiopathic PAH of 5.2% following THR and 2.3% following TKR.^20^ Meyer et al. described overall emergency and nonemergency perioperative mortality rates of 3.5%, and 2%, respectively, in an international prospective study evaluating the outcomes of patients with PAH undergoing either elective or emergency orthopedic, general, gynecological, or urology surgery.^14^ Price et al. demonstrated a 7% overall perioperative mortality rate in a single‐center retrospective study looking at the outcomes of patients with PAH presenting for orthopedic or general surgery, with all deaths following emergency surgery.^13^ Kaw et al. observed morbidity and mortality of PAH patients (with similar hemodynamics to the patients in the current study) undergoing surgery under general anesthesia to be 41% and 5%, respectively.^19^ Lai et al. reported an uncomplicated immediate postoperative course in patients with PH (diagnosed by echocardiography). However, patients had a higher mortality rate (9.7%) in the 30‐day postoperative period, with refractory heart failure being the leading cause of death.^17^ Finally, Ramkirishna et al. studied 145 patients with various types of PH who had undergone surgery under general anesthetic (80% being an emergency) and demonstrated an overall mortality rate of 7%, predominately due to right heart failure or respiratory failure.^15^

In our series, 72% of patients experienced one or more complications in the period between surgery and discharge. These are high event rates in comparison with elective arthroplasty surgery in patients without PH, as well as in comparison with previous studies of operative interventions in patients with PH.^13^, ^14^, ^15^, ^19^, ^20^, ^23^, ^24^, ^25^ Importantly no patients developed refractory acute heart failure or respiratory failure. The mean length of stay was 13 days, approximately 7–10 days longer than patients undergoing total hip or knee replacements in patients without PH.^26^, ^27^, ^28^, ^29^, ^30^, ^31^

### Patient selection and perioperative management

Patients were carefully selected having been established on PH therapy with hemodynamic improvement at repeat RHC and relatively well‐preserved RV function. Regional anesthesia has previously been associated with better outcomes than general anesthesia in patients with PAH.^13^, ^14^, ^16^ The transition from spontaneous breathing to intermittent positive pressure ventilation, the addition of positive end‐expiratory pressure, hypoxemia, hypercapnia, and sympathetic stimulation from laryngoscopy can all increase pulmonary vascular resistance and therefore RV afterload.^12^, ^16^ Furthermore, the majority of induction and maintenance anesthetic agents cause systemic vasodilation leading to a decreased mean arterial pressure.^16^ This can have severe consequences in a patient population with an already reduced functional cardiovascular reserve.^16^, ^32^, ^33^, ^34^, ^35^ For these reasons, surgery was carried out under regional anesthesia where possible. Where GA was necessary, we aimed to avoid intubation and fixed positive pressure ventilation if possible and used a SAD with spontaneous ventilation and pressure support at low pressures to control arterial carbon dioxide. For one patient, the anesthetist felt a SAD would not be appropriate. The lowest possible dose of propofol was used to avoid hypotension, facilitated by coinduction with a fentanyl dose of 1–2 μg/kg; this also helped to attenuate the sympathetic response to laryngoscopy and intubation.^16^ The vasodilation effects of both the regional and general anesthesia were treated by giving metaraminol, either with intermittent boluses or by starting a metaraminol infusion before the anesthetic was given, with the aim of maintaining the mean systemic blood pressure within its normal, preoperative range. High airway pressures can lead to an increase in pulmonary vascular resistance and are associated with patients with obstructive lung disease.^36^ Using pressure‐controlled ventilation can reduce the peak airway pressures, and therefore, was used in preference to volume‐controlled ventilation for the one intubated patient who had a background of bronchiectasis in addition to PH to avoid this risk. Each patient requires an individualized approach to airway maintenance and ventilation, to maintain the best oxygenation and least hypercapnia while using the lowest airway pressures possible. We used low‐dose intravenous iloprost in the majority of patients. Although chronic administration of intravenous prostanoids is associated with hemodynamic and prognostic improvements^37^, ^38^ the role of short‐term perioperative prostanoid therapy is not proven. It is possible that its use had a positive impact on outcomes, however, further study of its efficacy in this setting is required, especially with respect to its potential for reducing systemic vascular resistance and its antiplatelet effect. Of note, we did not observe any bleeding complications related to regional anesthesia in patients receiving iloprost.

All patients had an arterial line sited, which allowed both beat‐to‐beat monitoring of blood pressure, and in 86% of cases, LidCo was used for stroke‐volume optimization and goal‐directed fluid therapy. This allowed precise optimization of preload, which is particularly important in patients with RV hypertrophy and impairment. All patients had central venous catheters inserted before anesthesia, used to both monitor central venous pressures and central venous saturation as well as to be able to administer inotropic or vasoconstrictive medication in the case of deterioration.

Uncemented implants were used for all THR operations to eliminate the risk of bone cement implantation syndrome (BCIS); a rare, but important cause of perioperative mortality and morbidity in patients undergoing cemented hip arthroplasty. Cemented implants performed under an intraoperative tourniquet were used for all TKR operations, as BCIS appears to be a complication more commonly reported in THR than TKR.^39^, ^40^, ^41^, ^42^

All patients received Level 2 care and once our pathway was established, all patients were cared for by PH specialist with input from the orthopedic team. A key element was a multiprofessional team approach involving close cooperation between pulmonary vascular physicians, nurses, physiotherapists with a dedicated anesthetist, and orthopedic surgical team.

### Orthopedic and patient reported outcomes

All patients were documented as making “good progress” from an orthopedic perspective 6 weeks after their operation at a postoperative orthopedic review. OA and PH both adversely affect QoL and financial status.^5^, ^7^, ^43^, ^44^, ^45^, ^46^ We observed a significant improvement in QoL with a mean EmPHasis‐10 score reduction of 5.5 points (*p* = 0.04), consistent with a meaningful change.^47^, ^48^

In conclusion, by using a dedicated patient pathway and a multiprofessional approach we have demonstrated that carefully selected patients with PH can undergo elective lower limb orthopedic surgery with excellent outcomes, although with a higher perioperative complication rate and longer length of stay than in patients without PH.

### Limitation

This is a single‐center retrospective analysis involving a relatively small number of cases.

## CONFLICT OF INTERESTS

The authors declare that there are no conflict of interests.

## ETHICS STATEMENT

Ethical approval for this study was obtained from the NHS research and ethics committee (REC reference 16/YH/0352, IRAS project ID: 2114400).
